# Validation of Management Zones, Variability, and Spatial Distribution of the Physiological Quality of Soybean Seeds

**DOI:** 10.3390/plants14121856

**Published:** 2025-06-16

**Authors:** Maurício Alves de Oliveira Filho, Ana Laura Costa Santos, Ricardo Ferreira Domingues, Gabriela Mariano Melazzo, Brenda Santos Pontes, Rafael Jacinto da Silva, Sandro Manuel Carmelino Hurtado, Hugo César Rodrigues Moreira Catão

**Affiliations:** Instituto de Ciências Agrárias (ICIAG), Universidade Federal de Uberlândia (UFU), Uberlândia 38410-337, Brazil; mauricio.aof@gmail.com (M.A.d.O.F.); ana.santos7@ufu.br (A.L.C.S.); ricfdomingues@hotmail.com (R.F.D.); gabriela.melazzo@ufu.br (G.M.M.); brendinha_spontes@ufu.br (B.S.P.); rafaeljsilva@ufu.br (R.J.d.S.)

**Keywords:** *Glycine max* (L.) Merrill, precision agriculture, vigor, spatial variability, geostatistics

## Abstract

Precision agriculture facilitates improved management by studying the spatial and temporal variability of soil attributes. Soybean (*Glycine max* (L.) Merrill) seeds may exhibit distinct quality when produced in different management zones. This study aimed to validate management zones during seed production and identify the variability and spatial distribution of soybean seed physiological quality using geostatistical tools. Management zones were defined based on interpolated maps of soil and vegetation attributes using the Smart Map Plugin (SMP) within the QGIS environment. Post-harvest, the variability of physiological seed quality across different management zones was assessed. Germination, accelerated aging, dry weight, emergence, electrical conductivity, and tetrazolium tests were conducted in a completely randomized design. Soil attributes, initial plant stand, and soybean seed productivity validated the management zones. Physiological seed quality varies across the production field, particularly in terms of vigor, thereby enhancing diagnostics through map interpolation. Geostatistics enable determination of the spatial distribution of soybean seed physiological quality in seed production areas, facilitating decision-making regarding harvest zones.

## 1. Introduction

The desire for a more developed and sustainable world is a reality proposed by the United Nations (UN) through the creation of the 17 Sustainable Development Goals (SDGs), promoted by the Food and Agriculture Organization (FAO). The ability to manage agricultural environments based on spatial and temporal variation—precision agriculture (PA)—provides higher production, greater sustainability, and reduces negative environmental impacts, aligning with SDG 12: sustainable production and consumption [1].

The economic sector involving one of the world’s main commodities, soybeans (*Glycine max* (L.) Merrill), has been growing steadily over recent years. As a major global producer, Brazil has followed this growth, currently producing 155 million tons, the highest production recorded in a 10-year period [2]. The increasing production capacity is directly linked to technological advances in agriculture, with the application of good management practices and the use of high-quality seeds being among the key factors impacting the productive potential of the crop [3,4].

Soil, in its entirety, is a source of life for numerous macro and microorganisms, and it is a place for various physiological, metabolic, and physical interactions, making it a highly heterogeneous and complex environment. Precision agriculture has enabled better management practices based on the study of the spatial and temporal variability of soil attributes [5], allowing for the partitioning of this soil heterogeneity into homogeneous zones, also known as management zones (MZs). This approach has enabled the achievement of higher seed production potentials and greater profitability for producers [6].

Although there is potential to increase production using precision agriculture, the variability in soybean seed quality may differ according to different management zones [7]. By understanding the seed quality in relation to these zones, it would be possible to better target the harvest, making it more efficient and yielding batches with the highest quality standards. This would lead to profound changes in this operation, as the process would no longer be carried out uniformly, as it is today. Further changes could occur in other post-harvest stages, such as seed processing and storage, according to the quality variability.

Production fields are subject to various influences that can affect the final quality of the seeds produced. Among these are soil attributes, the occurrence of pests and diseases, water availability, and others. Therefore, seed producers require high-quality seed lots and need to manage the spatial variation in their production fields to meet the standards set by the Ministry of Agriculture (MAPA) [8].

The integration of precision agriculture techniques to evaluate the spatial distribution of quality components is essential for creating maps of physiological quality component spatialization, defining areas of high- and low-vigor seeds in the production field, and serving as an information management and decision-making tool [9]. Based on the above, the objective of this study was to validate the definition of management zones based on seed production and identify the spatial variability in the physiological quality of soybean seeds.

## 2. Material and Methods

**Figure 1:**
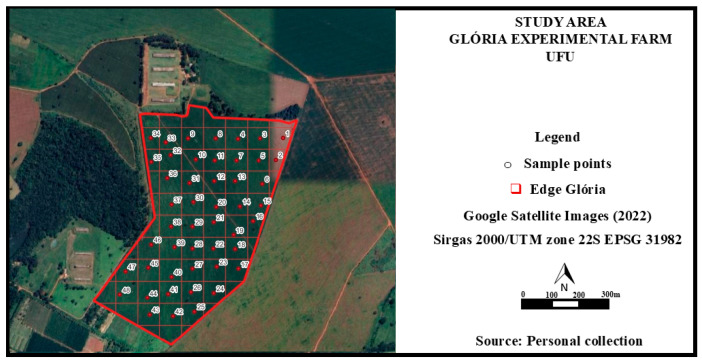
Location of the experimental area for soybean seed production and the grid of 48 sampling points in the 2022–2023 harvest.

The production of soybean seeds from the cultivar 75HO111 CI IPRO (commercial name: HO APORÉ IPRO) (HO Genética, Goiânia, Brazil) was conducted at the experimental farm of Glória, Universidade Federal de Uberlândia (UFU), Uberlândia campus, MG, Brazil (18°57′15.6″ S, 48°11′57.4″ W, 920 m altitude), in a 48-hectare area (Figure 1).

**Figure 2:**
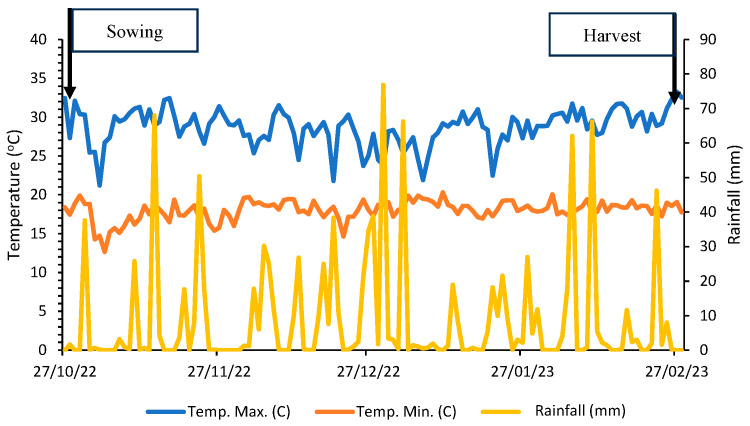
Precipitation (mm), maximum and minimum temperatures (°C) corresponding to the period from sowing on 28 October 2022 to harvest on 27 February 2023, during the soybean (*Glycine max* (L.) Merrill) seed production period used in the experiment.

In the region, the predominant climate is of the Aw type (tropical), characterized by hot and humid summers and predominantly dry winters [10]. The average annual rainfall is around 1500 mm, and the soil type is red latosol. The area’s history involves direct sowing in soil covered with crop rotation straw of sorghum, corn, and soybean under rainfed conditions. During seed production, environmental conditions were monitored via stations, with data on rainfall (mm) and maximum and minimum temperatures (°C) (Figure 2) collected through the UFU meteorological station, located at the experimental farm of Glória.

### 2.1. Data Collection of Soil and Vegetation Attributes for the Definition of Management Zones

Using the QGIS program [11], a regular, random, and systematic sampling grid with 48 points was established prior to sowing, with a sampling density of 1 point per hectare (Figure 1). The sampling points were identified using a GPS system (Garmin E-trex Vista^®^) (Olathe, KS, USA). According to the soil analysis methods manual, both disturbed and undisturbed soil samples were collected to characterize the attributes of the experimental area. These attributes included texture classification (sand and clay), soil electrical conductivity, organic matter, total cation exchange capacity (CEC), available phosphorus (P-Mehlich), penetration resistance, initial plant stand, and soybean seed productivity.

Disturbed Samples: Collected within a 5 m perimeter around each central point, 10 sub-samples were taken from a depth of 0–0.2 m and later homogenized into a single aliquot of 150 g to represent the sampling point.

Undisturbed Samples: A Kopecky ring was used to collect samples at a depth of 0–0.2 m at each sampling point. Each ring was labeled and stored, and sealed with plastic wrap.

Penetration Resistance Samples: Using Penetrolog^®^ equipment from Falker (Porto Alegre, Brazil) with a type 2 cone tip, 3 samples were collected from each sampling point at a depth of 0.2 m.

### 2.2. Crop Installation, Plot Marking, and Harvest

Sowing took place on 28 October 2022 at an average depth of 3 cm with a spacing of 0.5 m between rows, aiming for a population of 15 plants/m. At each of the 48 sampling points, experimental plots were marked with a useful area of 8 m^2^, consisting of 4 rows spaced 0.5 m apart and 4 m long, at the V1–V2 phenological stage of the crop. Area marking and initial plant stand evaluations were conducted for the points within the sampling grid.

The manual harvest of experimental plots was conducted on 27 February 2023, with seed moisture content of 13.1%, 12.3%, and 14.1% for the high-, medium-, and low-potential management zones, respectively. All plants from the experimental plots (8 m^2^) were harvested to determine productivity, followed by threshing. The weight of one thousand seeds was determined, with moisture adjusted to 13%, to calculate productivity.

### 2.3. Soybean Seed Quality Variability

Soybean seeds were first processed and then homogenized in a Johnes-type homogenizer with 18 channels. After this process, the seeds from the 48 sampling points underwent sieve retention using a set of 5 sieves with diameters of 7.0, 6.5, 6.0, 5.5, and 5.0 mm. Each sample was shaken for an average of one minute, selecting seeds retained on the 6.0 mm sieve for subsequent quality analyses.

The seeds from all sampling points were subjected to quality evaluation through the following tests.

Germination Test: Seeds were evenly distributed between two germination paper sheets, with distilled water applied at 2.5 times the dry weight of the paper. Four rolls containing 50 seeds each (200 seeds per treatment) were placed in plastic bags and kept in a B.O.D. chamber at 25 °C with a 12 h photoperiod. The normal seedlings were evaluated on the 8th day [12], and results are expressed as percentages (%).

Dry Weight: Performed on the normal seedlings from the germination test (8 days). Seedlings were dried at 65 °C for 72 h in a forced-air oven. After drying, the material was weighed on a precision scale [13]. Results are expressed in grams.

Accelerated Aging: Acrylic boxes with a metal screen were used for seed aging. Distilled water (40 mL) was added to each box, and seeds were placed on the screen in a single layer. The boxes were sealed and kept in a B.O.D. chamber at 41 °C for 48 h [14]. Four repetitions of 50 seeds were then subjected to the germination test, and normal seedlings were evaluated on the fifth day after sowing, with results expressed in percentages (%).

Emergence in Greenhouse: Four repetitions of 50 seeds were subjected to the emergence test on sand substrate in plastic trays. Seeds were evenly distributed and sown at a depth of 3 cm. Trays were placed in a greenhouse at ambient temperature, with two daily irrigations (morning and afternoon) with approximately 60% of the water retention capacity [13]. The evaluation was conducted on the 10th day after sowing. Normal seedlings were evaluated, with results expressed in percentages (%).

Electrical Conductivity: Seed vigor was assessed by determining the amount of solutes leached into the imbibition solution [15]. Four repetitions of 50 seeds were placed in disposable plastic cups (200 mL capacity), and the samples were weighed on a precision scale (0.001 g). After adding 75 mL of deionized water to each cup, the cups were kept in a B.O.D. chamber at 25 °C for 24 h in the dark. After imbibition, the solution was agitated and conductivity readings were taken using an MCA 150 conductometer, with results expressed in μS cm^−1^ g^−1^ of seed.

Tetrazolium Test: Initially, seeds were pre-conditioned in a humid atmosphere inside acrylic boxes containing 40 mL of distilled water. Seeds were placed in a single layer on a metal screen to standardize their moisture content. After 24 h in a B.O.D. chamber at 25 °C, the seeds were placed in germination paper packages, moistened with 2.5 times their dry weight, and placed in a Mangelsdorf germinator at 25 °C for 16 h. After pre-conditioning, seeds were divided into 4 repetitions of 25 seeds for each treatment in 40 mL plastic cups and submerged in a tetrazolium solution (0.075%). The seeds were kept in a B.O.D. chamber at 40 °C for 3 h. After staining, seeds were classified according to [16] into three vigor classes: Class 1 (high vigor: classes 1–3), Class 2 (low vigor: classes 4–5), and Class 3 (non-viable seeds: classes 6–8).

### 2.4. Definition of Management Zones (MZs)

In the QGIS environment with the Smart Map Plugin (SMP), the management zones (MZs) were defined based on the interpolation of attributes such as altitude, total sand, clay, soil electrical conductivity, potential CEC, organic matter, available phosphorus, penetration resistance, initial soybean plant stand, and soybean seed productivity. The steps for defining the MZ included selecting the analysis attributes, data interpolation, defining the ideal number of classes, and obtaining the final management zones map using the fuzzy K-means algorithm.

### 2.5. Statistical Analysis

Soil attributes and seed quality data were subjected to descriptive statistical analysis using Excel^®^ 365 version to calculate central tendency measures (minimum, maximum, mean, and median) and the coefficient of variation. Spatially, the data were analyzed using geostatistics, employing semivariogram models and kriging interpolation [17], with the aid of Surfer^®^ version 10 (Golden, CO, USA) [18] and QGIS version 3.28 [11] for the creation of thematic maps. In the absence of models, interpolation was performed using inverse distance weighted (IDW). The validation was carried out based on the analysis of the correspondence between the management zones and the physiological quality attributes of the seeds for each zone using the *t*-test (*p* < 0.05).

## 3. Results and Discussion

### 3.1. Creation of Semivariograms and Kriging Interpolation of Soil and Vegetation Attributes for the Development of Management Zones

Table 1 shows the descriptive statistics for the soil and vegetation attributes of the experimental area. By analyzing the mean and median of the attributes, it is possible to observe the proximity of the values. This indicates that the data follow a normal distribution [19]. The lowest coefficient of variation was found for the altitude attribute (1.0%), while the highest (C.V. = 58.2%) was observed for the P-Mehlich attribute.

After sampling and analyzing the soil attributes, a database was generated that enabled the interpolation of these attributes and the creation of spatial distribution maps with the aid of geostatistics. The creation and adjustment of models to the experimental semivariograms [17] were performed using the data from Table 2, with the assistance of Surfer10^®^ software [18].

There were differences in the model adjustments for the attributes, with the best fits tending towards the spherical model, which contrasts with the suggestions of [20,21], who recommend Gaussian model adjustments.

**Figure 3:**
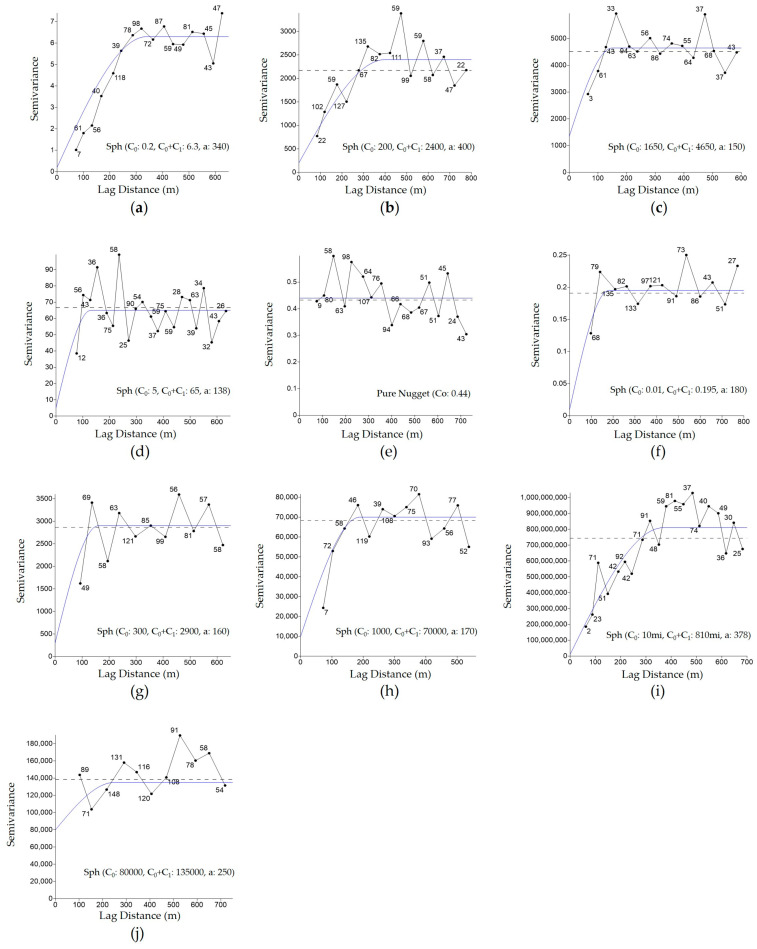
Semivariograms of the attributes used to define the management zones through Surfer^®^ software: altitude (**a**), total sand (**b**), clay (**c**), soil electrical conductivity (**d**), cation exchange capacity (**e**), soil organic matter (**f**), available phosphorus in soil (**g**), soil penetration resistance (**h**), initial stand plants (**i**) and soybean (*Glycine max* (L.) Merrill) seed productivity (**j**).

One of the main parameters of semivariograms (Figure 3) is the range (a), defined as the maximum distance at which sample points of a variable show spatial continuity (homogeneity) [22,23]. According to the data presented in Table 2, the range values varied from 138 to 400 m, with conductivity and sand showing the lowest and highest values, respectively. Therefore, it can be assumed that the grid of one point per hectare was adequate for data collection for mapping the variables, as the geostatistical models were adjusted with ranges (a) greater than 100 m.

According to [24], the soil organic matter variable, which showed a nugget effect (Co) close to zero, indicates low sampling error. [17] noted that the nugget effect is an important parameter in the analysis of attributes, as the greater the difference between Co and the plateau of the semivariogram (Figure 3), the more confidence can be placed in the estimate.

**Figure 4:**
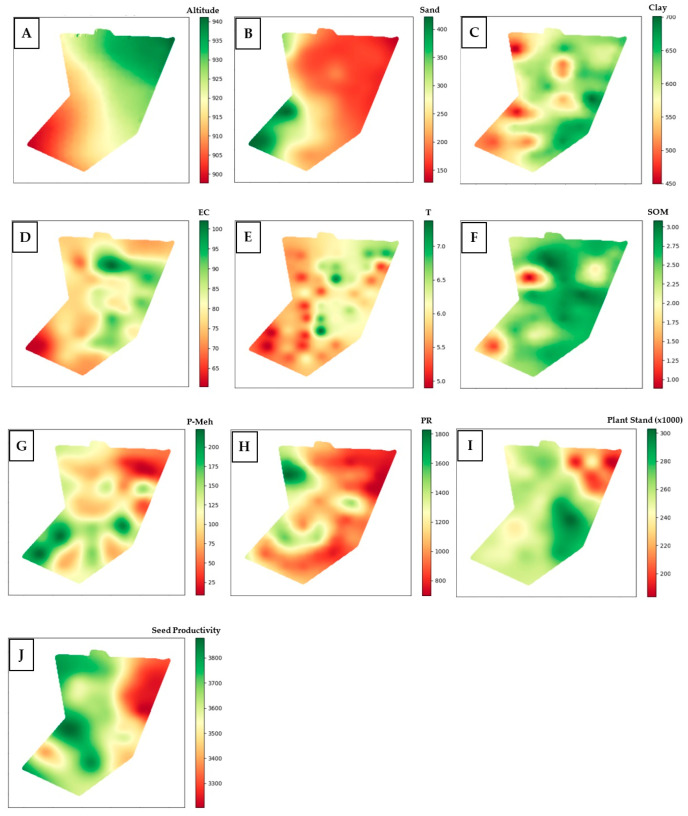
Spatial maps interpolated by kriging of soil attributes: altitude (**A**), total sand (**B**), clay (**C**), soil electrical conductivity (**D**), cation exchange capacity, IDW interpolation (**E**), soil organic matter (**F**), available phosphorus in soil (**G**), soil penetration resistance (**H**), and vegetation indices (soybean (*Glycine max* (L.) Merrill) initial plant stand (**I**), and soybean seed productivity (**J**), generated in the QGIS environment after semivariogram adjustments using Surfer^®^ software.

In the Qgis environment, along with the Smart Map Plugin (SMP), it was possible to interpolate each attribute, making it possible to gradually distinguish its expression in the experimental area through the generated maps. Kriging interpolation is an important tool for studying spatial behaviors, as seen in Figure 4.

In general, for the attributes of soil penetration resistance and organic matter content (Figure 4H and Figure 4F, respectively), homogeneous behaviors were observed, with resistance remaining at low levels and organic matter being present in high amounts across almost the entire experimental area. Analyzing Figure 4A–C, it is noticeable that the lower parts of the images on the left correspond to the areas with the lowest altitude of the experimental area. These areas are characterized by low clay content and high sand levels. In this same region, low soil electrical conductivity was observed (Figure 4D).

According to [25], soils with higher clay content have physical and chemical properties that contribute to better crop development, leading to higher productivity. In contrast, sandy soils have nutritional deficiencies that hinder optimal crop growth [26]. That said, in the spatially interpolated maps of Figure 4I (initial stand of soybean plants) and Figure 4J (soybean seed productivity), it was observed that the upper right region showed the lowest stand and productivity indices, indicating opposite behavior, as this region has low sand content and high clay content.

### 3.2. Definition of Management Zones in Soybean Seed Production Field

**Figure 5:**
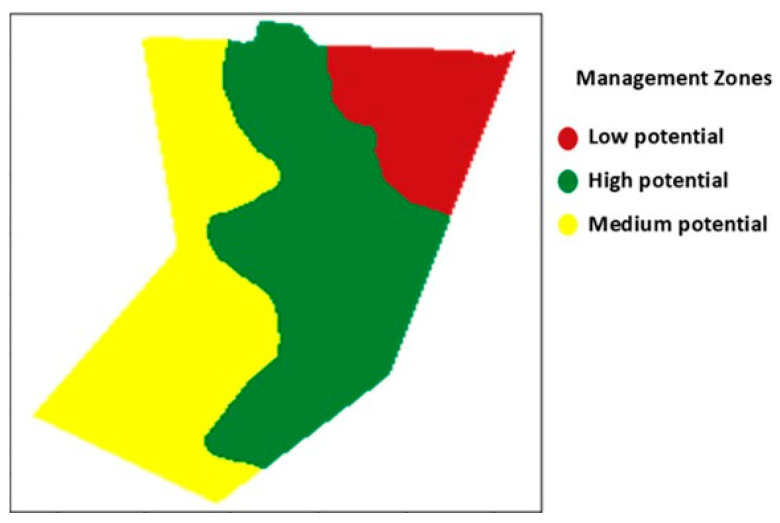
Map of management zones for the experimental area, highlighting high-potential zone (green), medium-potential zone (yellow), and low-potential zone (red).

It was possible to distinguish three management zones in the experimental area according to their potential, classifying them as high-, medium-, and low-productivity potential (Figure 5) based on attributes such as altitude, total sand, clay, soil electrical conductivity, potential CEC, soil organic matter, available phosphorus in the soil, soil penetration resistance, and vegetation indices (initial plant stand and soybean seed productivity). The literature often divides fields into different management zones using attributes such as sand and clay content, altitude, and productivity [27,28,29].

### 3.3. Variability in Soybean Seed Quality According to Management Zones (MZ)

In the analysis of the data described, it is possible to observe variations between the minimum and maximum values of the physiological seed quality attributes (Table 3).

This variation may have occurred due to the influence of biotic and abiotic factors, as well as the physicochemical aspects of the soil, hot climate, plant physiological stress, and the high rainfall index during the cultivation phase, all of which can impact soybean seed quality [30].

Upon examining the data, the lowest coefficients of variation were found for accelerated aging (5.1%) and emergence (10.5%). The highest coefficient of variation was observed for the tetrazolium class 3 data (39.3%) (Table 3). This could indicate drastic variations in the data due to the biotic and abiotic factors that occurred in the experimental area, leading to seed mortality. The values close to the mean and median suggest a behavior similar to normal distribution [19]. It is important to highlight the proximity of the values found for tetrazolium class 1 and tetrazolium class 2, as in these classes, soybean seeds are considered alive, differing in vigor [16].

**Figure 6:**
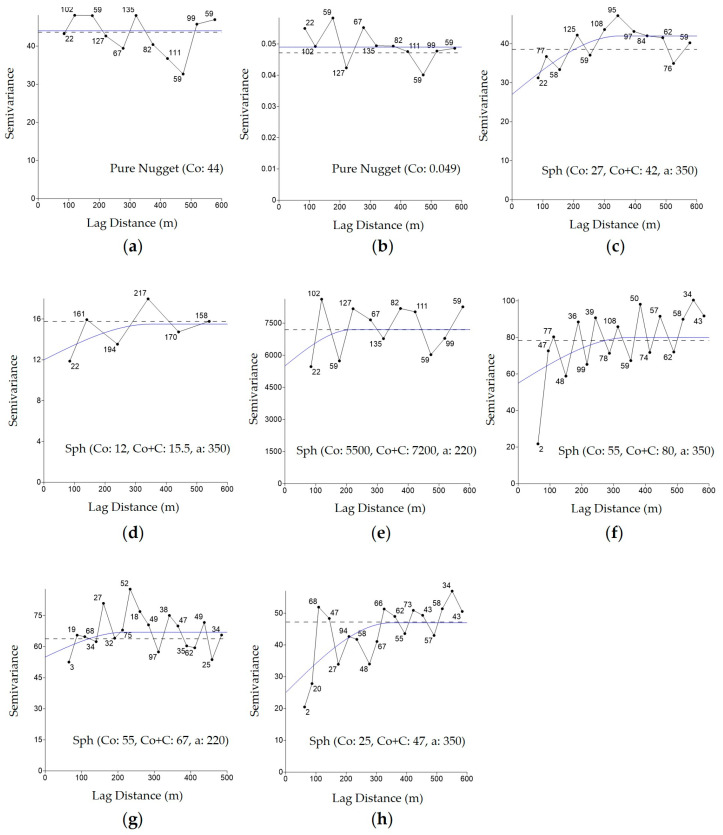
Semivariograms adjusted using Surfer^®^ software based on the results of the following tests: germination (**a**), dry mass (**b**), accelerated aging (**c**), emergence (**d**), electrical conductivity (**e**), tetrazolium class 1 (**f**), tetrazolium class 2 (**g**), and tetrazolium class 3 (**h**) of soybean (*Glycine max* (L.) Merrill) seeds and seedlings from different management zones.

Spatial behavior was observed for most of the attributes evaluated, as reported by [17]. The attributes of soybean seed quality evaluated through accelerated aging, emergence, electrical conductivity, and tetrazolium tests (classes 1, 2, and 3) showed semivariograms adjusted using the spherical model (Figure 6).

Germination and dry mass tests showed pure nugget effects, making it impossible to model semivariograms for these attributes. This may have occurred because the germination test was conducted under ideal conditions of light, temperature, humidity, and oxygen [12]. For the dry mass test, normal seedlings from the germination test were used. The absence of model adjustment means that there is independence between samples, as well as a spatial distribution occurring randomly. A future spatial dependence analysis for these attributes should consider a grid with more than 1 point per hectare.

Regarding the semivariograms, the range observed varied from 220 to 350 m, with the highest values for accelerated aging, emergence, tetrazolium class 1, and tetrazolium class 3. The range represents the maximum distance at which the samples are spatially correlated with each other [23]. Beyond this distance, the samples no longer showed spatial relationships.

**Figure 7:**
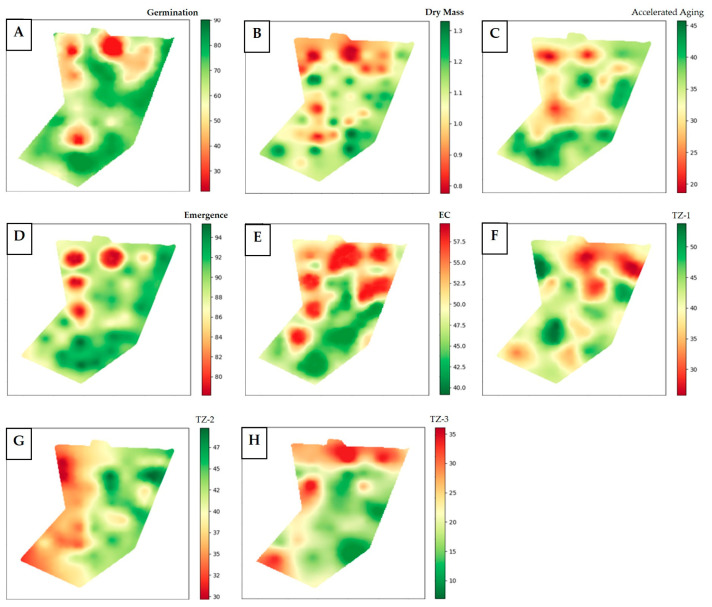
Spatial maps interpolated by kriging in the QGIS environment based on the results of germination tests: IDW interpolation (**A**), dry mass, IDW interpolation (**B**), accelerated aging (**C**), emergence (**D**), electrical conductivity (**E**), and tetrazolium classes 1 (**F**), 2 (**G**), and 3 (**H**) of soybean (*Glycine max* (L.) Merrill) seeds and seedlings from different management zones.

The interaction of the results from the spatial distribution of quality components associated with the georeferenced mapping allowed for the identification of regions with high- and low-vigor seeds within the seed production field. From the generated maps, it is possible to observe the spatial behavior of the physiological quality evaluation results of soybean seeds in different management zones (Figure 7).

The maximum germination of soybean seeds was 91%, meeting the minimum germination standard for commercialization (80%) established by legislation [31]. Germination varied by 74% across the different management zones. When analyzing the data from the germination test, a coefficient of variation of 17.6% was observed (Table 3). The initial quality of the seeds may have been strongly influenced by the climatic conditions before harvest due to the rainfall that occurred at the end of the production process, as shown in Figure 2. The main effects of the rain during the pre-harvest of soybean seeds include pod opening, which facilitates the proliferation of fungi; germination of seeds still within the pods; rotting, fermented, or moldy grains; pest attacks, such as stink bugs and beetles; and deterioration due to moisture. After physiological maturity, the seed is physiologically disconnected from the mother plant, facing exposure to environmental conditions that may be unfavorable [32].

In the tetrazolium class 3 test, the highest coefficient of variation (39.3%) was observed, indicating that viability is more sensitive to environmental variations. According to [33], during the seed deterioration process, the loss of the ability to germinate is the last step before seed death, while vigor tests are based on events that happen earlier.

By analyzing the interpolated maps of germination, accelerated aging, dry mass, emergence, and tetrazolium class 1 (Figure 7), it is possible to observe that the upper parts of the maps are colored red, indicating lower results, which justifies a direct relationship between these attributes. Similarly, high values (red coloring) of electrical conductivity were observed in the same areas of production with lower-quality seeds. This indicates a greater release of intracellular contents from the seeds [34], i.e., a higher level of deterioration is observed in this location based on the map interpolation. The region with the highest emergence presented lower electrical conductivity, meaning the seeds produced in this region tend to have higher physiological quality. Ref. [8] observed a similarity between the maps of emergence, accelerated aging, and electrical conductivity.

According to [35,36], vigor tests allow for a better diagnosis of seed physiological quality since they are more sensitive in detecting deterioration. Through the study of spatial variability and mapping, it is possible to distinguish with greater precision the regions within the production field that contain seeds with superior quality.

From the maps generated based on the tetrazolium data, it is possible to observe that in the zone characterized as low potential (Figure 5), fewer seeds classified as class 1 (high vigor) were produced (Figure 7F). In the high-potential zone, an inverse relationship was observed, producing fewer seeds classified in class 3 (non-viable seeds) (Figure 7H). In class 3, a high incidence of damage from moisture, mechanical damage, and stink bugs was found, leading to soybean seed death. High levels of stink bug and moisture damage are directly related to the reduction in soybean seed vigor [16]. By spatializing the tetrazolium test results, it is possible to highlight the distribution of these damages in the production area, enabling the identification of regions with higher intensity [9,37].

Even with all the tools available for crop management aimed at maximizing productivity—and in the case of seeds, high physiological quality—this is not always possible. Despite all the available technology, the amount of seeds from certain regions has been severely compromised due to high levels of deterioration caused by moisture, stink bug injuries, ruptured seed coats, and mechanical damage [35].

With this in mind, we carried out the validation of the management zones in contrast with the physiological quality of the seeds and observed that the high-potential zone exhibited seeds with higher quality through germination, electrical conductivity, and a lower percentage of seeds in class 3 of the tetrazolium test. Seeds produced in the low-potential zone showed lower germination, higher electrical conductivity, and a lower percentage of seeds in class 1 of the tetrazolium test. No differences were observed between the management zones and seed physiological quality through accelerated aging, dry mass, and emergence tests (Table 4).

Geostatistics allow for determining the spatial distribution of the physiological quality of soybean seeds in the production area, facilitating decision-making about areas to be harvested [36]. The maps obtained through data interpolation serve as a seed quality management tool, allowing for the definition of areas to be harvested or discarded within a seed production field [35,38].

Thus, through geostatistics and the variability in soybean seed quality, more accurate decisions can be made, particularly regarding harvest direction, to obtain seed lots with high quality.

## 4. Conclusions

The analyzed attributes allow the validation of management zones based on the quality attributes of soybean seeds.

The physiological quality of the seeds is not uniform across the production field, especially regarding vigor, providing a better diagnosis through map interpolation.

Geostatistics enable the determination of the spatial distribution of the physiological quality of soybean seeds in seed production areas, facilitating decision-making regarding areas to be harvested.

## Figures and Tables

**Table 1:** Descriptive statistics for the evaluated soil and vegetation attributes.

Attributes ^1^	Minimum	Maximum	Mean	Median	* C.V. (%)
Altitude (m)	901.3	937.8	921.5	921.7	1.0
Clay	393.8	739.9	593.4	611.0	12.6
EC	60.2	106.9	81.2	79.0	11.5
T	4.8	7.9	5.9	6.0	12.4
P-Mehlich (mg/dm^3^)	5.3	255.6	109.3	93.2	58.2
PR	670.4	1921.1	1100.2	1024.8	28.8
SOM	0.7	3.1	2.5	2.6	18.6
Initial plant stand (plants/m^2^)	165,000.0	311,000.0	254,125.0	255,000.0	11.5
Soybean seed productivity (kg/ha)	2560.6	4500.0	3580.6	3604.6	11.0

* C.V. (%): coefficient of variation. ^1^ EC: soil electrical conductivity; T: cation exchange capacity; P-Mehlich: available phosphorus in soil; PR: penetration resistance; SOM: organic matter in soil.

**Table 2:** Geostatistical analyses for the evaluated soil and vegetation attributes.

Attributes ^1^	Model	Nugget (C_0_)	Partial Sill (C_1_)	Sill (C_0_ + C_1_)	Range (a)
Altitude	Spherical	0.2	6.1	6.3	340
Clay (20 cm)	Spherical	1.350	3.300	4650	150
Sand (20 cm)	Spherical	200	2200	2400	400
EC (20 cm)	Spherical	5	60	65	138
T (20 cm)	Pure Nugget	0.44	--	--	--
P-Mehlich (20 cm)	Spherical	300	2.600	2.900	160
PR (20 cm)	Spherical	1.000	60.000	70.000	190
SOM (20 cm)	Spherical	0.01	0.185	0.195	180
Initial Plant Stand (plants/m^2^)	Spherical	10,000,000	800,000,000	810,000,000	378
Soybean Seed Productivity (kg/ha)	Spherical	80.000	55.000	135.000	250

^1^ EC: soil electrical conductivity; T: cation exchange capacity; P-Mehlich: available phosphorus in soil; PR: penetration resistance; SOM: organic matter in soil.

**Table 3:** Descriptive analysis based on the results of germination tests, accelerated aging, dry mass, emergence, electrical conductivity, and tetrazolium test classes of soybean (*Glycine max* (L.) Merrill) seeds and seedlings from different management zones.

Physiological Attributes (n = 48)	Minimum	Maximum	Mean	Median	C.V. (%)
Germination (%)	17	91	73	79	20.1
Accelerated Aging (%)	3	59	34	34	41.6
Dry Mass (g)	0.3	1.5	1.0	1.1	21.7
Emergence (%)	48	98	89	91	10.5
E. Conductivity (μS cm^−1^ g^−1^)	25.5	88.2	49.2	46.1	26.0
Tetrazolium 1 (%)	19	66	40	40	22.7
Tetrazolium 2 (%)	21	60	40	40	22.2
Tetrazolium 3 (%)	6	40	19	18	39.3

C.V. (%): coefficient of variation, in %.

**Table 4:** Validation of the management zones based on the analysis of correspondence between the zones and the physiological quality attributes of soybean (*Glycine max* (L.) Merrill) seeds.

Management Zones	G (%)		AA (%)		DM (g)		E (%)		EC (μS cm^−1^ g^−1^)		TZ1 (%)		TZ2 (%)		TZ3 (%)	
Low potential	71	B	36	a	1.0	a	90	a	55.3	a	37	b	41	a	22	a
Medium potential	71	B	35	a	1.0	a	89	a	49.8	ab	45	ab	34	b	21	a
High potential	80	A	36	a	1.1	a	91	a	44.0	b	40	b	44	a	16	b

Means followed by the same letter in the column do not differ based on *t*-test (*p* < 0.05). G: germination; AA: accelerated aging; DM: dry mass; E: emergence; EC: electrical conductivity; TZ1: tetrazolium class 1; TZ2: tetrazolium class 2; TZ3: tetrazolium class 3.

## Data Availability

Data is contained within the article.
